# Structured morphological modeling as a framework for rational strain design of *Streptomyces* species

**DOI:** 10.1007/s10482-012-9760-9

**Published:** 2012-06-21

**Authors:** Katherine Celler, Cristian Picioreanu, Mark C. M. van Loosdrecht, Gilles P. van Wezel

**Affiliations:** 1 Molecular Biotechnology, Institute of Biology, Leiden University, PO Box 9505, 2300 RA Leiden, The Netherlands; 2 Department of Biotechnology, Delft University of Technology, Julianalaan 67, 2628 Delft, The Netherlands

**Keywords:** Morphological modeling, Fermentation, Microscopy, Enzyme, Antibiotic, SsgA

## Abstract

Successful application of a computational model for rational design of industrial *Streptomyces* exploitation requires a better understanding of the relationship between morphology—dictated by microbial growth, branching, fragmentation and adhesion—and product formation. Here we review the state-of-the-art in modeling of growth and product formation by filamentous microorganisms and expand on existing models by combining a morphological and structural approach to realistically model and visualize a three-dimensional pellet. The objective is to provide a framework to study the effect of morphology and structure on natural product and enzyme formation and yield. Growth and development of the pellet occur via the processes of apical extension, branching and cross-wall formation. Oxygen is taken to be the limiting component, with the oxygen concentration at the tips regulating growth kinetics and the oxygen profile within the pellet affecting the probability of branching. Biological information regarding the processes of differentiation and branching in liquid cultures of the model organism *Streptomyces coelicolor* has been implemented. The model can be extended based on information gained in fermentation trials for different production strains, with the aim to provide a test drive for the fermentation process and to pre-assess the effect of different variables on productivity. This should aid in improving *Streptomyces* as a production platform in industrial biotechnology.

**Electronic supplementary material:**

The online version of this article (doi:10.1007/s10482-012-9760-9) contains supplementary material, which is available to authorized users.

## State of the art in growth modeling of filamentous microorganisms

Streptomycetes are Gram-positive mycelial bacteria which are commercially used in the production of natural products such as antibiotics, anticancer agents and immunosuppressants, as well as industrial enzymes (Hopwood 2007). Unlike unicellular bacteria, which grow exponentially by binary fission with a constant generation time (Errington et al. 2003), filamentous organisms grow due to the combination of steady hyphal growth and addition of new hyphal tips via branching of the mycelium. During growth, vegetative hyphae are divided into compartments by cross-walls (Chater and Losick 1997). The reproductive phase is initiated by the erection of sporogenic structures called aerial hyphae, which are nonbranching structures that differentiate following a complex cell division event whereby the multigenomic hyphae are converted into chains of unigenomic spores. Aerial hyphae are formed only on solid-grown cultures, giving the colonies their characteristic white and fluffy appearance. Some *Streptomyces* species are also able to produce spores in submerged culture (Glazebrook et al. 1990; Kendrick and Ensign 1983).

Morphology and structure formation vary from species to species, based on strain-specific genetic make-up that is yet poorly understood (Jakimowicz and van Wezel 2012). Many genes and physiological mechanisms are involved in the development of a particular morphological type (Kossen 2000). A survey of the submerged growth of over 100 reference species identified a continuum of morphological types, ranging from large macroscopic mycelial pellets several millimeters in diameter to small fragmented particles (Tresner et al. 1967). The different types of mycelia have been classified as pellets (compact masses of over around 1 mm in diameter), clumps (less compact masses between 0.6 and 1 mm in diameter), branched hyphae and non-branched hyphae (Pamboukian et al. 2002). *Streptomyces* species can be further subdivided into those which sporulate in liquid culture (*S.* *albus, S. griseus, S. roseosporus*), and those that do not (Glazebrook et al. 1990; Kendrick and Ensign 1983; van Wezel et al. 2009). When grown under different conditions, growth rate and morphology change depending on the composition of the growth medium, pH, temperature, mixing intensity, dissolved oxygen concentration and inoculum (Tough and Prosser 1996; Cui et al. 1998). In a sense, mycelial morphology is the classic example of “nature versus nurture”—an observed morphology emerges from the combination of genetic and environmental factors in a fermentation.

The filamentous nature of streptomycetes, resulting in highly viscous broths, unfavorable pellet formation and slow growth, strongly affects the rheology of liquid cultures, which makes fermentation difficult (van Wezel et al. 2009). Large clumps are mainly physiologically active around the edge of the pellet, with oxygen and nutrient depletion in the centre. Increased broth mixing may improve transport, but results in shearing off of pellet tips and lysis. Shear force may also rupture the pellet as a whole, especially if the pellet is already hollow due to oxygen or substrate limitations (Meyerhoff et al. 1995). In addition, downstream processing of fermentation broths is complex and costly (van Wezel et al. 2006). The understanding and control of morphology is therefore key for optimization of industrial fermentations. The relationship between growth and morphology, on the one hand, and biomass accumulation and productivity on the other, is complicated, and optimal morphology varies from product to product.

Studies on erythromycin production by *Saccharospolyspora erythraea* showed a strong correlation between mycelium fragment diameter (defined as the minimum diameter of a sphere that can bound a *hyphal fragment*, or pellet) and productivity, with a critical pellet diameter of 88 μm, below which production was drastically reduced (Wardell et al. 2002). Variants with decreased branching frequency showed increased hyphal strength, larger mycelial fragments, and increased antibiotic production. In *Streptomyces,* regulation of the secondary metabolism is complex, necessitating directed systems-level engineering approaches for strain improvement (van Wezel and McDowall 2011). One of the most direct ways of tackling the morphological problems was achieved by overexpression of the SsgA protein, which results in fragmentation of mycelial clumps (Kawamoto et al. 1997; van Wezel et al. 2000a). SsgA and its paralogue SsgB are required for the activation of cell division in streptomycetes, with SsgB recruiting the cell division scaffold protein FtsZ (Keijser et al. 2003; Willemse et al. 2011), and the enhanced expression of SsgA improving growth rates in batch fermentations of *Streptomyces coelicolor* and *Streptomyces lividans,* and resulting in a two-fold increase in yield of enzyme production with a higher production rate (van Wezel et al. 2006). Secretion capacity is also directly related to the activity of SsgA (Noens et al. 2007). This highlights the potential of genetic engineering approaches based on understanding of the biological processes that govern morphology and production. The effects of enhanced division on antibiotic production are less predictable (van Wezel et al. 2000b), and better insight into this relationship is needed.

Morphological modeling is a valuable tool to suggest potential strain improvements and predict optimal fermentation conditions. Present models largely investigate the influence of environmental factors on morphology, while modeling with a strong focus on genetics may be powerful (Kossen 2000), but has not been attempted. Several models for growth of filamentous organisms (both fungal and actinomycete) have been proposed, including single-pellet models that focus on microscopic morphology and model three-dimensional tip elongation and branching. An initial model was based on diffusion–reaction of a hypothetical intracellular growth-limiting component (Yang et al. 1992a); this model was later extended to include the diffusion of limiting substrates and fragmentation due to shear forces (Meyerhoff et al. 1995). Basing growth on a tip extension rate depending on oxygen concentration, a similar model combined microscopic morphology with analysis of solute profiles along the pellet radius and the fractal dimension (Lejeune and Baron 1997). Macroscopic (fermentation) models focus instead on the effects of mass transport on growth and production in a reactor, providing e.g. an unstructured approach for modeling growth of mycelial pellets in submerged cultures. These models integrate growth kinetics at hyphal scale with the physical mechanisms of mass-transfer processes in pellets and the fermentor (van Suijdam et al. 1982). Modeling of microbial kinetics may also be based on structured models that describe rates by means of selected cell components rather than by the undifferentiated biomass (Nielsen and Villadsen 1992). Fermentation-scale models are typically combined with a population balance, in which the behavior and effect of pellet populations in cultivations is studied, e.g. predicting changes in the distribution of pellet sizes within a population growing in a fermentor (Tough and Prosser 1996). Fermentation population models have also been coupled with structured models, with a particular focus on morphological forms, with various growth and production rates in different hyphal elements. In an early example of structured modeling, hyphae of *Aspergillus awamori* were divided into five differentiation states with different growth and metabolite synthesis rates (Megee et al. 1970). This approach was extended to include fragmentation and compared to experimental data for submerged growth of *Geotrichum candidum*, *Streptomyces hygroscopicus*, and *Penicillium chrysogenum* (Nielsen 1993). Population-based structural models were used to study the production of among others penicillin (Birol et al. 2002) or streptomycin (Liu et al. 2005). While these models keep track of the proportions of hyphal elements in a fermentor, they do not incorporate changes to the developing pellet during the fermentation process. Each structural element represents a fraction of the clump, without providing insight into the three-dimensional pellet morphology. Despite the strong increase in computational power and available modeling software, little has been done in recent years to improve and expand these models.

In this work growth of a mycelial pellet of *Streptomyces* is modeled, combining three-dimensional morphological pellet formation with a structured approach. We propose a combined morphological and structured model of a single-pellet, with a three-dimensional computational framework including oxygen (or other solute) diffusion and reaction in pellets, hyphal growth, branching and shearing, cross-wall formation and fragmentation, as well as collision detection during development. Biological information regarding the processes of differentiation and branching in liquid cultures of the model organism *S. coelicolor* has been implemented. The current modeling platform allows for study of the relationship between enzyme or antibiotic production and morphology and structure.

## Mathematical model description

**Fig. 1:**
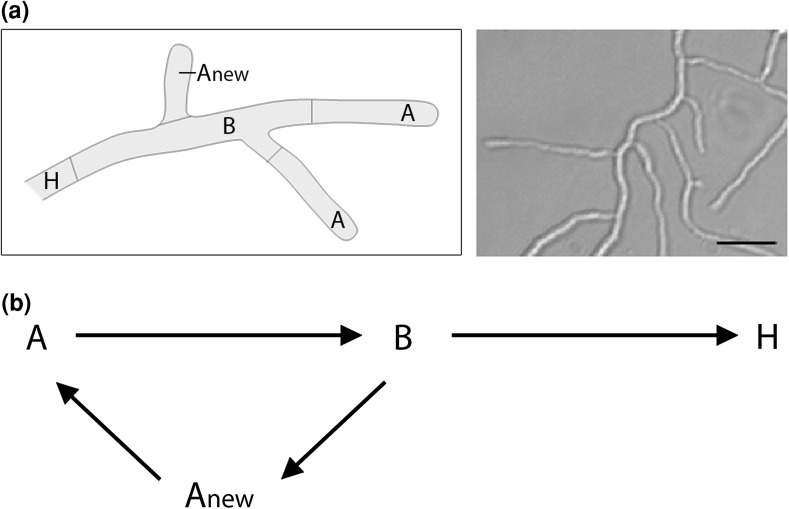
Graphical representations of hyphal growth**. a** *Left*: Hyphal element with different compartments: apical (*A*), subapical (*B*), newly-formed apical as a result of branching (*A*_new_) and hyphal (*H*). *Right*: light microscopy image of *S. coelicolor* hyphae for comparison. *Scale bar*, 3 μm. **b** Metamorphosis reactions between different hyphal compartments: apical (*A*), subapical (*B*), newly-formed apical as a result of branching (*A*_new_) and hyphal (*H*)

Fermentations involve many interacting factors of biological, chemical and physical nature. The microorganism itself, and its genetic make-up, are the backbone of the process, but process conditions (nutrients, oxygen, heat, mixing) also play a crucial role in controlling microbial growth, hyphal/pellet morphology and productivity. Mathematical models should incorporate these variables, so as to provide a *test drive for the fermentation process* and to pre-assess the effect of different variables on productivity. Although all cells in a hyphal element share a common cytoplasm with multiple nuclei, significant cellular and functional differentiation within the mycelial pellet exists (Megee et al. 1970). Earlier studies indicate that secondary metabolite production in filamentous organisms is associated with this morphological differentiation, which favors a structured approach to modeling (Giudici et al. 2004; Manteca et al. 2008). In creating a structured model, hyphae may be divided into three compartments: (i) apical, (ii) subapical, and (iii) hyphal compartment, each type indicating a different stage of cellular differentiation (Fig. 1a).

At the start of growth, a spore germinates to form a new apical compartment (A_new_). Oxygen and substrate are assimilated within the apical compartment for growth and apical extension (Gray et al. 1990). The apical compartment A extends until a maximum apical length is reached. As apical extension continues, some of compartment A is converted into subapical compartment B. Compartment B has an intracellular composition very similar to compartment A. However, oxygen and substrate consumption in compartment B results not in growth, but rather in the formation of new branches (A_new_). Within mycelial pellets, where substrate levels are depleted, compartment B transforms into the hyphal compartment H. Based on the observation that pellet and clump formation are important determinants for yield, it is the hyphal compartment that is taken to be responsible for secondary metabolite production (Manteca et al. 2008). The various metamorphosis reactions described are shown in Fig. 1b.

### Growth

To describe three-dimensional growth of the mycelium, a hyphal tip growth and branching model (Yang et al. 1992a) was adapted, with the assumptions that hyphae are cylindrical, have constant diameter *d* and density *ρ*_*x*_, and grow by apical extension. Three-dimensional collision detection is employed to prevent overlapping hyphae. The orientation of the growing tip is characterized by angles θ and φ in spherical coordinates that change stochastically as a function of time. During growth, an apical compartment extends until the maximum apical length (*L*_*A,max*_) is reached, which may occur before the formation of a cross-wall near the tip. Tip extension is exponential until *L*_*A,max*_ is reached, after which apical extension continues, but rather than extending compartment A, a new compartment B is added. In this case, the net result of tip extension is actually the formation of subapical cells from apical cells.

Growth rate is based on local oxygen concentration according to Monod kinetics (with maximum specific growth rate μ_*max*_ and half-saturation coefficient *K*_*O*_). The total growth rate in a shell with thickness *dr* situated at distance *r* from the pellet centre is calculated as the sum of extension rates α of all tips in that shell (Eq. (1)). The total growth rate is correlated with the oxygen consumption rate by a yield coefficient *Y*_*OX*_.

1 $$ \mu (r)C_{X,A} (r) = \frac{1}{V}\left( {\frac{{\pi d^{4} }}{4}} \right) \cdot \frac{{C_{O} (r)}}{{K_{O} + C_{O} (r)}} \cdot \sum {\alpha_{i,\max (A)} } $$

Although *Streptomyces* grow by apical extension, the model assumption is that once the apical compartment has reached its maximum length, growth results in formation of subapical compartment Eq. (2).

2 $$ \mu (r)C_{X,H} (r) = \frac{1}{V}\left( {\frac{{\pi d^{4} }}{4}} \right) \cdot \frac{{C_{O} (r)}}{{K_{O} + C_{O} (r)}} \cdot \sum {\alpha_{i,\max (B)} } $$

Ageing cells become increasingly vacuolated and have a completely different metabolism than actively growing apical cells (Zangirolami et al. 1996). Differentiation is the process of conversion of subapical cells B to hyphal cells H once a certain arbitrary differentiation age (*A*_*diff*_) has been reached. The assumption is made that at this time point, growth results in formation of hyphal compartment Eq. (3), reflecting the natural differentiation observed, but still poorly understood, within *Streptomyces sp.* Hyphal cells do not grow or branch, but are responsible for secondary metabolite production. The amount of hyphal cells in a pellet can be taken as indicative of the level of secondary metabolite production.

3 $$ \mu (r)C_{X,H} (r) = \frac{1}{V}\left( {\frac{{\pi d^{4} }}{4}} \right) \cdot \frac{{C_{O} (r)}}{{K_{O} + C_{O} (r)}} \cdot \sum {\alpha_{i,\max (H)} } $$

Because of the hyphal differentiation assumed in the model, apical, subapical and hyphal compartments may have different oxygen consumption rates. However, because it is not known whether this is a valid assumption, a single yield coefficient is taken for all compartments. Upon fitting the model to experimental data, different yield coefficients may be obtained.

### Branching

New branches form by the extension of new tips A_new_ from the subapical compartment B. These tips grow in the plane perpendicular to the parent segments. Initially, a new coordinate system is set up with the parent branches defining the x–y plane, and a z-vector drawn perpendicular to the plane. The direction of the new branch growth in this plane is chosen stochastically from a uniform distribution. Once the branch endpoint has been chosen within the new coordinate system, this point is placed back within the original coordinate system. In *Streptomyces*, branching depends on the essential protein DivIVA, which localizes at tips and new branch sites, recruiting or activating cell wall synthesis enzymes (Flärdh 2003; Hempel et al. 2008). In the model, branching occurs according to the local oxygen concentration in the pellet: probability of branching tapers off towards the inside of the pellet, where the oxygen concentration is diminished. As branching rates have not been measured within pellets, the correlation of branching and oxygen concentration is a model assumption made to decrease levels in the crowded inner core of a pellet. Distance between branches results from the branching probability and a chosen branching interval (*b*_*int*_). As in growth, collision detection is employed to prevent overlapping hyphae during branching.

### Cross-wall formation

The current model relies on the assumption that cross-walls form near branches (Reichl et al. 1990). Once cross-wall formation has been initiated, a cross-wall will form directly before or after a branch. Subsequent cross-walls on the branch will form at a multiple (random uniform distribution) of the specified interval (*c*_*int*_) from the first cross-wall. Cross-wall formation occurs at a chosen time interval. Multiple cross-walls may simultaneously form on a given branch during a cross-wall formation event, as evidenced in live-imaging experiments (Jyothikumar et al. 2008). The number of cross-walls formed on a given hyphae is dependent on the number of branches on the hyphae. However, if the choice of new cross-wall position is at another branch point or location of an existing cross-wall, the cross-wall is not formed.

### Fragmentation

Stirring in the fermentor creates shear forces that enforce fragmentation of the pellets. Similarly to the model of Meyerhoff, a biomass density parameter (m^3^ biomass/m^3^ total volume) was chosen above which hyphae are not affected by shear because they stabilize each other within the pellet. A respective threshold radius (*r*_*thres*_) is chosen based on this density, and a breaking probability (*P*_*break*_) is chosen at a certain distance to this radius from the tip (*r*_*tip*_). The expression for the probability of breaking, assuming shear force parameter *λ*_*shear*_, is given by Eq. (4).

4 $$ P_{break} = 100.0 - 100.0 \cdot exp\left[ { - \lambda_{shear} \cdot \frac{{(r_{tip} - r_{thres} )}}{{r_{thres} }}} \right] $$

Breaking is assumed to occur at cross-wall locations, where the hyphal wall is reported to be weaker (Krabben and Nielsen 1998), and this is supported by the strong effect of the cell-division activator protein SsgA on fragmentation (Traag and van Wezel 2008).

### Collision detection

The computational framework includes collision detection between the hyphal branches, which has not been incorporated in any previous model for micro-scale pellet formation. Collision detection is required to build a realistic model, as it avoids overlap of growing and branching hyphae in a spatially constrained environment. Moreover, the implemented collision detection algorithm ensures that the resulting pellet volume densities do not surpass 100 % filled volume near the pellet centre. Diffusion of oxygen and substrates into the pellet can then be based on real rather than hypothetical pellet cell volume density. Further, data on the location of e.g. the cell division and secretion machineries along the hyphae can be implemented in the model.

In the collision detection algorithm, the space domain is partitioned into cubes and all segments of the mycelium are placed in the space cube corresponding to their location. Collision is checked between a new hyphal segment and other segments in the same and neighboring space cubes. A collision is detected if any points on the segments are closer than the distance of two hyphal radii from each other. The algorithm from (Ericson 2005) was used for determining the distance between two segments. Once a near collision has been detected, tip growth or branching does not take place in the given time step. In the following time step, should a growth or branching angle be stochastically chosen which does not result in collision, extension of the mycelium at the given location can take place. This corresponds to observations during live imaging of hyphal growth which revealed that when two hyphal tips are found close to each other, one may exert apical dominance over the other and arrest the latter’s growth for a period of time [(Jyothikumar et al. 2008) and our unpublished data].

### Oxygen diffusion

Oxygen is taken to be the limiting substrate, based on previous studies which have shown that oxygen can become mass transfer limited within mycelial pellets (Michel et al. 1992). The one-solute assumption is made here for the simplicity of the case study and more solutes can easily be taken into account. When a pellet reaches a certain critical size, oxygen limitation within the centre occurs, resulting in lysis. This critical size is a function of the pellet biomass density, the dissolved oxygen concentration in the bulk liquid and the hyphal respiration rate (Cui et al. 1998).

Initially, a fully three-dimensional (3D) model for the oxygen diffusion and reaction within the pellet was developed. Oxygen concentrations in concentric shells obtained with the three-dimensional model were subsequently compared with the radial (one-dimensional, 1D) oxygen profile obtained assuming spherical symmetry (Lejeune and Baron 1997). Obviously, the 3D model was computationally much more intensive than the 1D counterpart, but nevertheless resulted in a very similar oxygen concentration profile. Therefore, the simplified 1D radial symmetry was further assumed for each pellet, with oxygen transport occurring by molecular diffusion. Alteration in local oxygen concentration is assumed to be slow compared to growth, and hence a differential equation with stationary coupling of oxygen diffusion and reaction rates can be written in radial coordinates *r* Eq. (5).

5 $$ D_{O2,eff} \frac{1}{{r^{2} }}\frac{d}{dr}\left( {r^{2} \frac{{dC_{O} }}{dr}} \right) = \frac{\mu (r)}{{Y_{XO} }}C_{X} (r) = OUR $$

The expression for oxygen uptake rate (*OUR*) can be rewritten to take into account the extension of the apical, subapical or hyphal compartment at different points in the branch lifetime Eq. (6–8). The apical compartment is extended if the branch length is less than the maximum apical compartment length *(L*_*branch*_ < *L*_*A,max*_ *.)* The subapical compartment is extended thereafter until the branch reaches differentiation age (*A*_*diff*_), at which point the hyphal compartment is extended. Until data is obtained to prove otherwise, the yield coefficients of biomass on oxygen *Y*_*XO,A*_, *Y*_*XO,B*_, *Y*_*XO,H*_ are assumed to be the same in all compartments.

6 $$ OUR = \frac{\mu (r)}{{Y_{XO,A} }}C_{X,A} (r)\quad {\text{if }}L_{branch} < L_{A,\max } $$

7 $$ OUR = \frac{\mu (r)}{{Y_{XO,B} }}C_{X,B} (r)\quad {\text{if }}A_{branch} < A_{diff} $$

8 $$ OUR = \frac{\mu (r)}{{Y_{XO,H} }}C_{X,H} (r)\quad {\text{otherwise}} $$

Within the pellet, the effective diffusion coefficient *D*_*eff*_ was computed proportional to the pellet porosity (ε). The boundary conditions were (1) constant oxygen concentration in the bulk phase and (2) zero oxygen flux (symmetry condition) at the center of the pellet.

### Parameter values and simulation

The computational model was implemented in MATLAB (MATLAB 2008b, Mathworks, Natick, MA, www.mathworks.com) and visualization of pellets was performed using the freeware Persistance of Vision Raytracer software (Pov-Ray, www.povray.org). The 1D diffusion–reaction solution was approximated using the finite differences method. Parameter values for a typical simulation run are given in Table 1. Parameters for variation in tip angle direction are based on measurements performed in growth chambers (Yang et al. 1992b). Branching and cross-wall intervals are parameters that are strain dependent; differences in the intervals result in significant morphological variation. The yield of biomass on oxygen was based on literature, with a value of 1.2 kg kg^−1^ assumed for all compartments (Meyerhoff et al. 1995). The model may later be fitted to experimental data to determine whether different yield coefficients exist for each compartment type. The shearing probability was adjusted to result in realistic breakage.

**Table 1:** Values of model parameters used in a typical simulation run for pellet morphology

Parameter	Unit	Value	Source
r	μm	0.25	*S. coelicolor* average radius
ρ_x_	kg · dw m^−3^	100	Assumed (Lejeune and Baron 1997)
α_max_	μm h^−1^	5	Conservative estimate
dθ	°	12	(Yang et al. 1992b)
dφ	°	12	(Yang et al. 1992b)
b_int_	Branch μm^−1^	1/2	Estimated
c_int_	Cross-wall μm^−1^	1/10	Estimated
D_O2_	m^2^ s^−1^	2.25 × 10^−9^ (at 30 °C)	Diffusion coefficient of oxygen in water
K_O2_	kg m^−3^	1.0 × 10^−4^	(Meyerhoff et al. 1995)
Y_XO_	kg kg^−1^	1.2	(Meyerhoff et al. 1995)
C_b,O2_	kg m^−3^	0.009	(Lejeune and Baron 1997)
λ_shear_	–	0.5	(Meyerhoff et al. 1995)

## Model output

The results of a simulation can be assessed by visualizing the mycelium morphology development (growth, branching and cross-wall formation) or numerical analysis of several quantitative measures. These measures include: the hyphal growth unit (HGU), fractions of different mycelial types over time or space, number of tips for a given morphology, number of cross-walls formed, biomass density, number of fragmentation events, etc. Here, we will limit the discussion to visualization, the HGU and component fractions.

### Visualization of pellet development

**Fig. 2:**
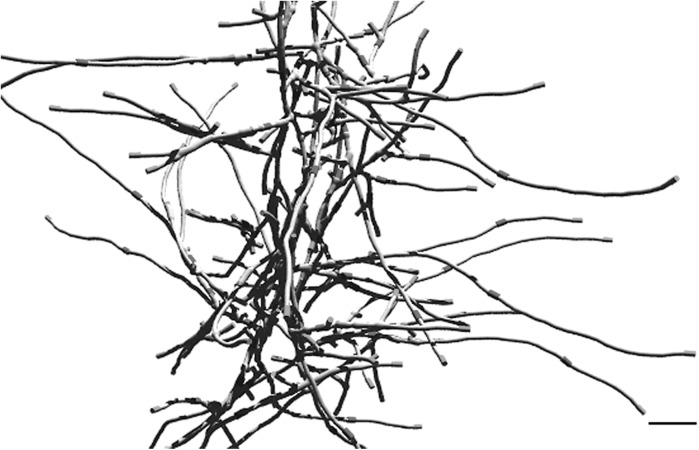
Simulation of early mycelial pellet formation with DivIVA localizing at hyphal tips (*green*) and cross-walls (*red*) forming at roughly 10 μm intervals. *Scale bar*, 5 μm

Modern software enables first-rate visualization of biological information, such as the localization of cell division proteins within developing hyphae. Morphological differentiation of streptomycetes is closely integrated with fundamental growth and cell-cycle processes (Flärdh and Buttner 2009). Implementation of knowledge on components that control morphogenesis (such as cell division components) or product formation (e.g. biosynthesis and secretion of natural products) is required to allow for building a more realistic model. A 3D rendering of a simulated portion of a growing mycelium is given in Fig. 2. The DivIVA protein (marked as green) drives tip growth and branching and is therefore always present at apical sites (Hempel et al. 2008). Cross-walls, where cell division proteins localize, are given in red. The potential of modern visualization software to enable realistic rendering of hyphal growth and pellet formation is demonstrated. Protein localizations were derived from the in vivo localizations of GFP-tagged proteins. The model can be readily extended with novel biological data and insights, such as the localization of antibiotic and protein secretion machineries depending on growth or the function of novel morphoproteins.

### Hyphal growth unit

The ratio between the size of the mycelium and the number of tips is a characteristic morphological variable called the HGU of a simulated pellet (Caldwell and Trinci 1973). Although originally defined as the total mycelium length divided by the number of tips, it may also be based on the total mycelium volume or mass (Nielsen 1993). In this study, HGU is calculated according to the original definition based on the total mycelium length. If the HGU is constant, both tip extension rate and branching frequency are proportional to the specific growth rate of the biomass. The HGU also provides an idea of the mycelium morphology: a large value indicates long hyphal threads with few branch points, whereas a small value indicates a dense hyphal structure with many branch points (Nielsen and Villadsen 1992). The HGU provides a quick assessment of morphological type; a change of parameters which results in an increase in the HGU may provide a wider or more fragmented morphology with enhanced mass transfer capability and better performance in the fermentor.

### Ratio of compartment types

Given that secondary metabolite production in filamentous organisms is associated with morphological differentiation in the mycelium, it is interesting to compare the ratio of different compartment types over time for a given morphology. Recent structured models correlate the amount of antibiotic production in a fermentation to the amount of subapical or hyphal compartments, where secondary metabolite formation is expected to take place (Paul and Thomas 1996; Birol et al. 2002; Giudici et al. 2004; Liu et al. 2005). The ratio of component types can be followed over pellet development, or alternatively, represented as a function of pellet radius. Classification of the mycelium into components with different metabolic activity and function may provide more understanding of the relationship between morphology and biomass accumulation and productivity.

## Case study

**Fig. 3:**
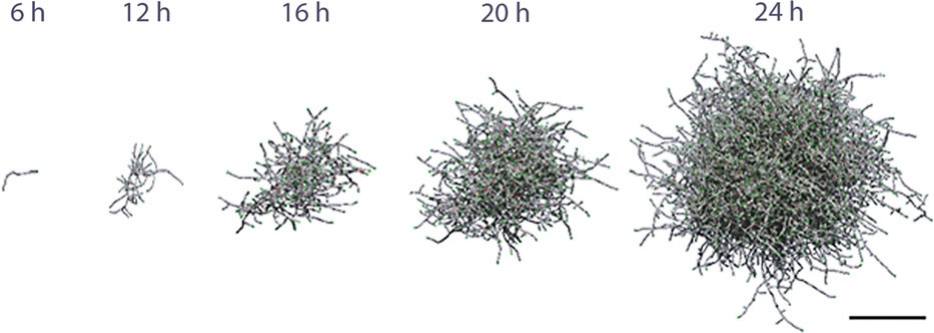
Two-dimensional projection of a 3D simulated pellet. Images based on the three-dimensional model presented as Supplemental Video. Parameters as given in Table 1. Growth time indicated. *Scale bar*, 100 μm

**Fig. 4:**
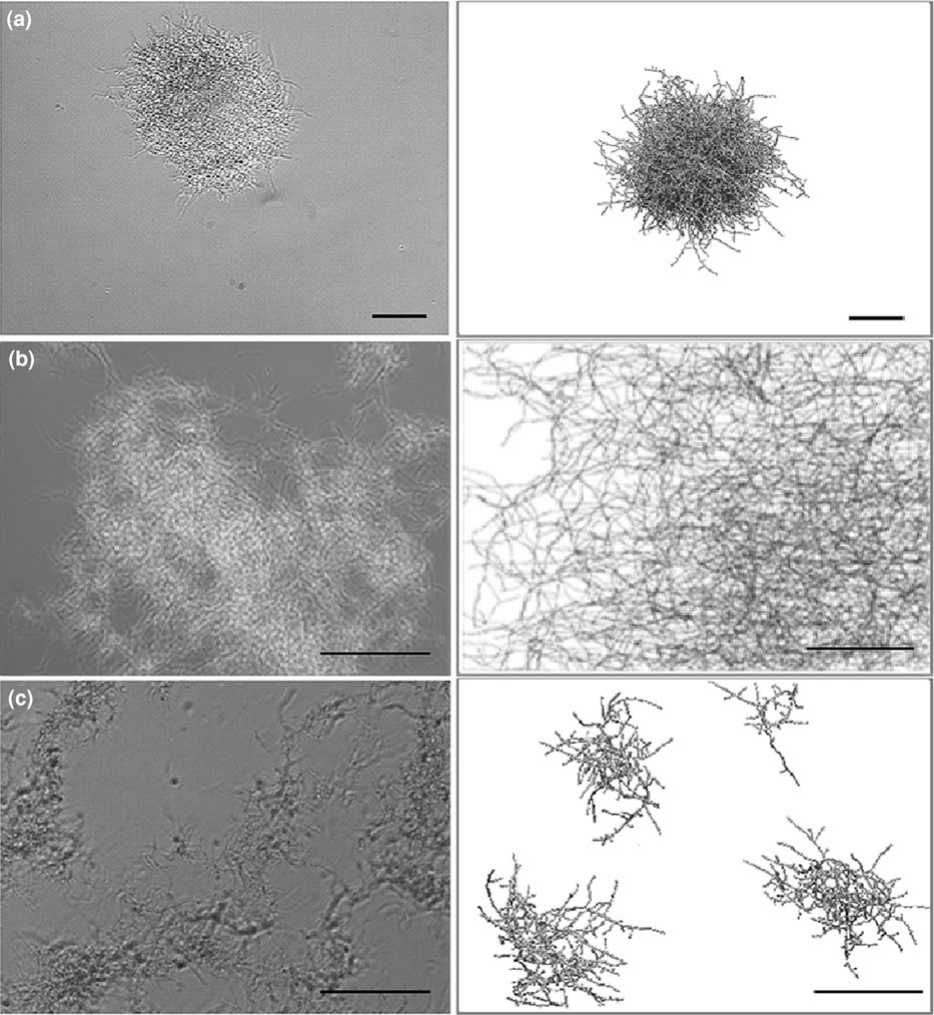
Qualitative comparison of real (*left*) and simulated (*right*) mycelial morphologies. Shake flask cultures were grown in TSBS/YEME medium at 30 °C for 20 h. **a** characteristic pellet produced by *S. coelicolor* M145 (wild-type strain); **b** mycelial mat produced by *S. lividans* variant MR (GPvW, unpublished); **c** fragmented growth of *S. coelicolor* GSA2 [overexpressing *ssgA*; (van Wezel et al. 2000a)]. For simulation parameters see Table 1. In case **b** branching interval was set to 1 branch/20 μm. In case **c** broken mycelial fragments are shown from a case where shear is taken into consideration. All the *bars*: 50 μm

**Fig. 5:**
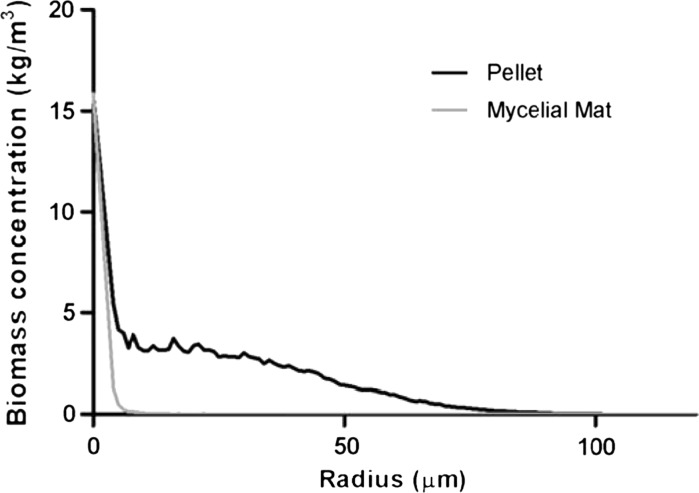
Biomass density profiles (kg biomass/m^3^ of pellet volume) for the pellet and mycelial mat morphologies showing increased density at the pellet/mat core and the larger density of the pellet morphology

A case study was performed to demonstrate the model’s ability to accurately show differences in *Streptomyces* strain morphologies and incorporate molecular information. Depending on model parameters the model represents the different morphological variants (pellets, mycelial mats or hyphal fragments). Quantitative model output parameters, such as the HGU and fractions of different component types are discussed. To visualize growth, an example of mycelium development over time is given (Fig. 3 and Supplemental Video). Starting from a single unbranched mycelium, a full quasi-spherical pellet with high inner cellular density and a loose outer layer of outgrowing filaments develops after 1 day. Different modeled morphological types were simulated and compared to real mycelial clumps with the described morphologies (Fig. 4). The biomass density profiles (kg biomass/m^3^ of pellet volume) for the pellet and mycelial mat morphologies are given in Fig. 5, showing the larger density of the pellet morphology. Pellet growth was simulated using the parameters given in Table 1; mycelial mat formation was simulated by increasing the distance between branches (from one branch every 2 μm to one every 20 μm); fragments were created when the pellet scenario was simulated taking shear into consideration. Formation of fragments (Fig. 3c) is representative of strains such as the *S. coelicolor* GSA2 strain over-expressing SsgA (van Wezel et al. 2000a), which shows increased sensitivity to shear and a pronounced tendency to fragment (van Wezel et al. 2006). Mycelial mat formation occurs naturally in other stains, such as *S. lividans* variant MR (GPvW, unpublished). Changing a single parameter value may have a major impact on the predicted morphology. By performing parameter sensitivity analysis studies and investigating the results, optimal production strains can be designed.

**Fig. 6:**
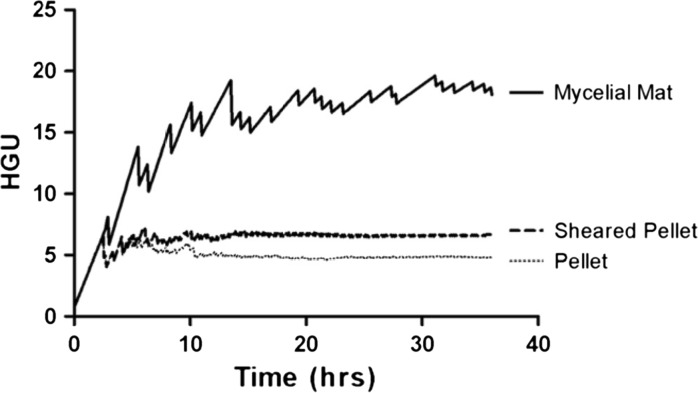
HGU values for simulated dense pellet, mycelial mat and sheared pellet morphologies over time

The HGU values were determined for the different simulated morphologies over 36 h of growth (Fig. 6), with higher HGU values indicating more open morphological structures. As expected, the dense pellet HGU is much smaller than that of the mycelial mat morphology. Fragmentation results in an increase in the HGU. Interestingly, for the pellet and sheared pellet (fragmented) morphologies, HGU levels off at roughly 12 h, while it continues increasing for the mycelial mat morphology. This indicates that in the mat, the mass of the mycelium increases more by extension of existing branches than by addition of new ones.

**Fig. 7:**
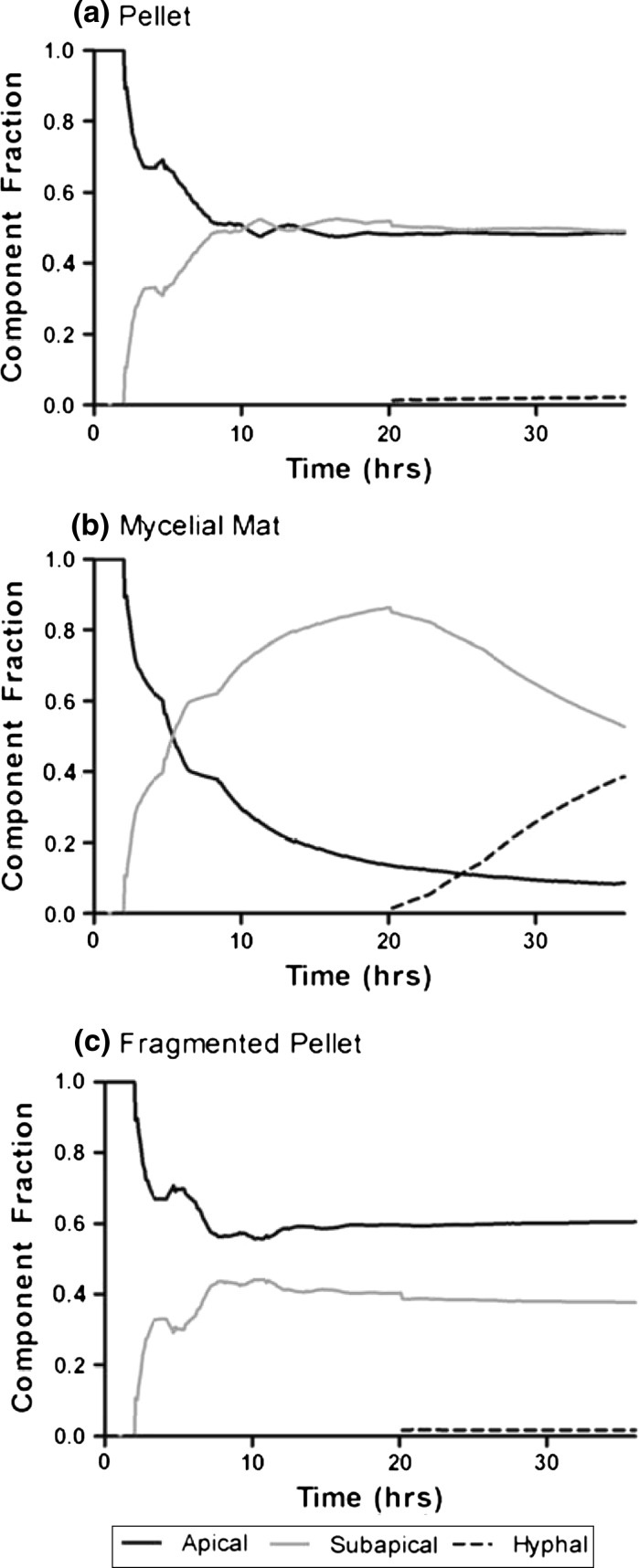
Comparison of component fractions (apical, subapical and hyphal) in simulated mycelial morphologies: **a** pellet, **b** mycelial mat, **c** fragmented pellet, plotted over time

Component fractions for the three simulations were compared (Fig. 7). In the pellet (Fig. 7a), dense growth of branches results in almost equal distribution of apical and subapical component at 36 h; ageing of cells, however, has begun. The hyphal fraction will extend steadily as oxygen and space limitations within the pellet limit growth and addition of new branches. In the mycelial mat (Fig. 7b), because of the reduced frequency of branching, at 36 h, the mycelium already consists of 40 % hyphal fraction and only 10 % apical component. Conversely, fragmented growth (Fig. 7c) results in a high apical fraction, due to the presence of a large new population of apical pieces.

In the above examples, the exact fraction values are not of importance. Rather, the plots serve to illustrate the potential of structural modeling, where the mycelium is considered to be a differentiating species, consisting of cells with different metabolic function. Such modeling can have an important role in the strategic genetic and morphological design of *Streptomyces* for industrial fermentations. As was demonstrated previously (Liu et al. 2005), fermentation trials can be used to modify the described morphogenesis reactions between structural components (Fig. 1b) such that the fractions of components directly correlate to production of metabolites.

**Fig. 8:**
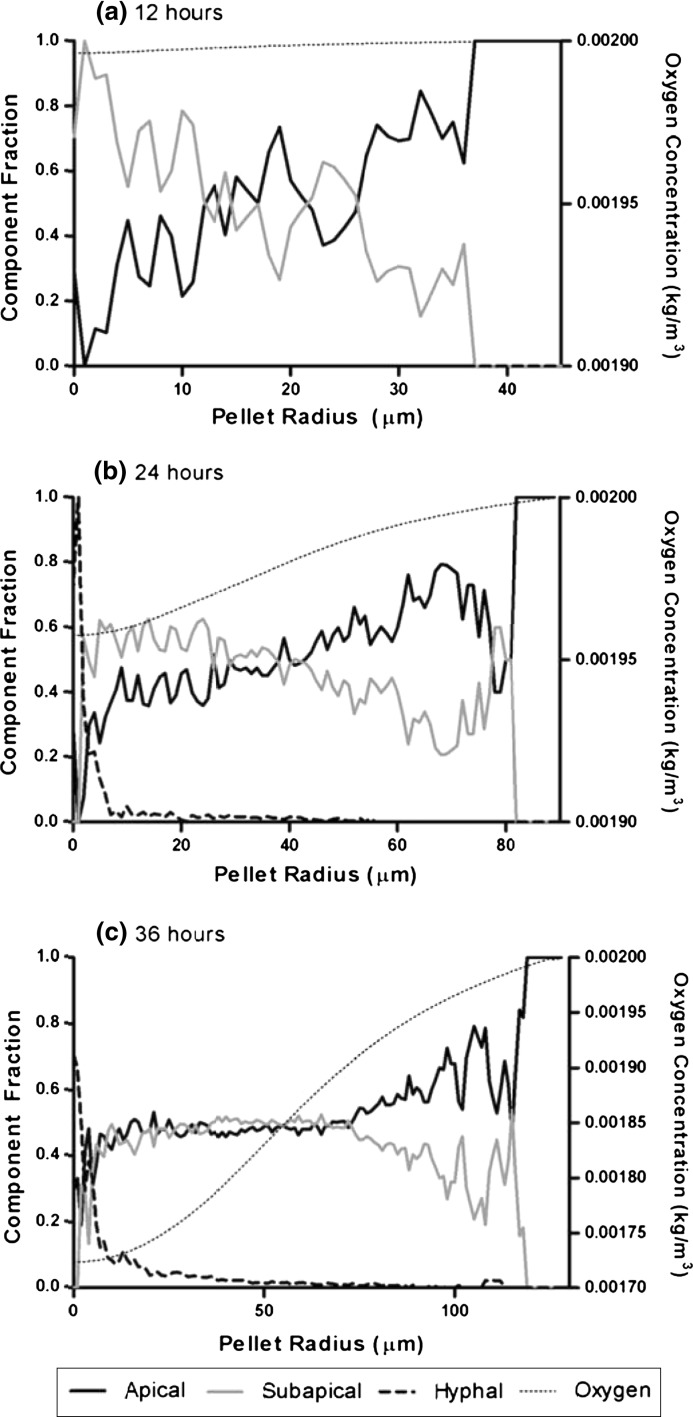
Fractions of apical, subapical and hyphal components versus pellet radius in a simulated dense pellet after **a** 12, **b** 24, and **c** 36 h of growth. Oxygen concentration along the radius indicates how branching frequency is affected by oxygen level. The hyphal compartment is located within the pellet core

The distribution of component fractions can also be plotted over hyphal radius at different time points (Fig. 8). The hyphal component is situated within the pellet centre because as the pellet ages, subapical compartment transforms into hyphal compartment. It is evident that oxygen limitation within the pellet results in decreased formation of branches, with an equal distribution of apical and subapical compartments. Where the oxygen level is higher, fraction of subapical component decreases because branching rate is higher and more apical compartments are being formed. The exterior of the pellet consists entirely of new, apical compartments. When coupled with confocal microscopy (Manteca et al. 2008) and microsensor measurements of oxygen concentration (Hille et al. 2005), such modeling can provide insight into processes that occur within pellets during fermentations.

## Conclusions and perspectives

The presented model integrates for the first time three-dimensional morphological visualization with structured modeling. It thereby provides a realistic, single-pellet, three-dimensional framework to study the relationship between enzyme or antibiotic production and morphology and structure. Simulations can be analyzed via visualization of mycelium development (growth, branching and cross-wall formation) or analysis of numerical measures, such as the HGU or fractions of different mycelial types over time or space. The output thus provides both a visual and numerical assessment of morphological type; for example, a change of parameters which results in an increase in the HGU may provide a wider or more fragmented morphology with enhanced mass transfer capability. Classification of the mycelium into compartments with different metabolic activity and function can provide better understanding of the relationship between morphology and biomass accumulation and productivity. The purpose of this type of modeling is to replace the conventional ‘black-box’ approach to morphological engineering with a directed rational design and evolution approach in order to better understand how growth rate and morphology affect secretion and yield. Literature describes key regulators that govern pivotal processes during growth in submerged culture, such as Crp for germination (Piette et al. 2005), DivIVA for tip growth and branching (Hempel et al. 2008) and SsgA for fragmentation (Kawamoto et al. 1997; van Wezel et al. 2000a), but further insight into these regulatory mechanisms is needed. Optimal morphology should facilitate fermentations from an engineering perspective as well as result in sufficient production of the desired metabolite product.

## Electronic supplementary material

Below is the link to the electronic supplementary material.

Supplementary material 1 (DOC 25 kb)

Supplementary material 2 (MP4 5042 kb)

## Variables

*A*_*branch*_: Branch age (h)
*A*_*diff*_: Differentiation age (h)
*b*_*int*_: Branch formation interval (m^−1^)
*c*_*int*_: Cross-wall formation interval (m^−1^)
*C*_*O*_: Oxygen concentration in fermentation broth (kg/m^3^)
*C*_*X*_: Concentration of biomass (kg/m^3^)
*C*_*X,A*_: Concentration of biomass, apical (kg m^−3^)
*C*_*X,B*_: Concentration of biomass, subapical (kg m^−3^)
*C*_*X,H*_: Concentration of biomass, hyphal (kg m^−3^)
*d*: Hyphal diameter (m)
*dt*: Time step (h)
*D*_*O2,eff*_: Effective diffusion coefficient for oxygen (m^2^ h^−1^)
*HGU*: Hyphal growth unit
*K*_*O*_: Microbial saturation coefficient for oxygen (kg m^−3^)
*L*_*A,max*_: Maximum length of apical compartment (m)
*OUR*: Oxygen uptake rate (kg m^−3^ h^−1^)
*P*_*branch*_: Probability of branching (% h^−1^)
*P*_*break*_: Probability of breaking (% h^−1^)
*r*: Radius of pellet (m)
*r*_*tip*_: Distance from pellet centre to tip (m)
*r*_*thres*_: Threshold radius (m)
*V*: Hyphal segment volume (m^3^)
*Y*_*XO*_: Yield of biomass on oxygen (kg kg^−1^)
*Y*_*XO,A*_: Yield of biomass on oxygen, apical (kg kg^−1^)
*Y*_*XO,B*_: Yield of biomass on oxygen, subapical (kg kg^−1^)
*Y*_*XO,H*_: Yield of biomass on oxygen, hyphal (kg kg^−1^)

## Greek symbols

*α*_*i,max*_: Maximum linear apical extension rate of branch *i* (m h^−1^)
*α*_*i,max(A)*_: Maximum linear extension rate of branch *i*, apical compartment (m h^−1^)
*α*_*i,max(B)*_: Maximum linear extension rate of branch *i*, subapical compartment (m h^−1^)
*α*_*i,max(H)*_: Maximum linear extension rate of branch *i*, hyphal compartment (m h^−1^)
*λ*_*shear*_: Shear force parameter
μ: Maximum specific hyphal growth rate (h^−1^)
*ρ*_*x*_: Density of hyphae (kg dw m^−3^)
*θ*: Polar angle in spherical coordinates
*φ*: Cone angle in spherical coordinates
