# Supplementary material for: Structured morphological modeling as a framework for rational strain design of Streptomyces species
Source: Antonie Van Leeuwenhoek. 2012 Jun 21;102(3):409–23. doi: 10.1007/s10482-012-9760-9 (PMC3456926; doi:10.1007/s10482-012-9760-9)
Supplement: Supplementary file 1 — Supplementary material 1 (DOC 25 kb) [file 10482_2012_9760_MOESM1_ESM.doc]

**Supplemental Material**

belonging to the manuscript *“Structured Morphological Modeling as a Framework for Rational Strain Design of Streptomyces species”*, by

Katherine Celler^1^, Cristian Picioreanu^2^, Mark C.M. van Loosdrecht^2^ and Gilles P. van Wezel^1,#^

**Affiliations:** ^1^ Molecular Biotechnology, Leiden Institute of Chemistry, Leiden University, PO Box 9502, 2300 RA Leiden, The Netherlands; ^2^ Department of Biotechnology, Delft University of Technology, Julianalaan 67, 2628 BC Delft, The Netherlands

# Author for correspondence. Tel: +31 71 5274310; Email: g.wezel@chem.leidenuniv.nl.

**Supplemental Video.**

Modeling of a *Streptomyces* pellet formed over time in submerged culture. The model represents 24 h of growth, with pellet diameter in the final frame roughly equal to 200 μm. Simulation parameters are given in the text in Table 1. The pellet was rendered using the Persistance of Vision Raytracer software (Pov-Ray, www.povray.org) and images were stitched together using QuickTime *(*www.apple.com/nl/quicktime/download)*.*
